# Dr. Major S. Langs

**Published:** 1916-02

**Authors:** 


**Dr. Major S. Langs** died at his home in Niagara Falls Jan. 20, aged 81. He was born in Langford, Ont., and came to the village of Suspension Bridge, now incorporated in the city of Niagara Falls, 47 years ago. He had held several offices, including that of trustee in the former village of Suspension Bridge, and was a prominent Mason. He had retired from practice and was a large property owner.

